# The Prepared Tau Exon-Specific Antibodies Revealed Distinct Profiles of Tau in CSF of the Patients with Creutzfeldt-Jakob Disease

**DOI:** 10.1371/journal.pone.0011886

**Published:** 2010-07-29

**Authors:** Cao Chen, Qi Shi, Bao-Yun Zhang, Gui-Rong Wang, Wei Zhou, Chen Gao, Chan Tian, Guo-Yong Mei, Yan-Ling Han, Jun Han, Xiao-Ping Dong

**Affiliations:** State Key Laboratory for Infectious Disease Prevention and Control, National Institute for Viral Disease Control and Prevention, Chinese Center for Disease Control and Prevention, Beijing, People's Republic of China; Ohio State University, United States of America

## Abstract

**Background:**

The diagnostic value of CSF tau for Creutzfeldt-Jakob disease (CJD) has been widely evaluated, showing a markedly disease-relative manner. However, the profiles of tau isoforms in CSF of CJD patients remain unknown. Here, we prepared the exon-specific antibodies against the peptides encoded by exon-2, exon-3 and exon-10 of human tau protein and evaluated the reactive profiles of tau in CSF samples from the patients with probable CJD.

**Methodology/Principal Findings:**

Sequences encoding exon-2, exon-3 and exon-10 of human tau protein were cloned into a prokaryotic expression vector pGEX-2T. Using recombinant fusion proteins GST-E2, GST-E3 and GST-E10, three tau exon-specific antibodies were elicited. Reliable specificities of the prepared antibodies were obtained after a serial of purification processes, not only in recognizing the tau peptides encoded by exon-2, -3 and -10, but also in distinguishing six recombinant tau isoforms by Western blot and ELISA. Three predominant tau-specific bands were observed in CSF samples with the exon-specific and the commercial tau antibodies, respectively, showing different reactive profiles between the groups of probable CJD and non-CJD. A 65 KD band was detected only in the CSF samples from probable CJD patients, especially with the antibodies against exon-2 (Anti-tE2) and exon-10 (Anit-tE10). The appearances of 65 KD band in CSF correlated well with positive 14-3-3 in CSF and typical abnormality in EEG. Such band was not observed in the CSF samples of six tested genetic CJD patients.

**Conclusions/Significance:**

Three exon-specific polyclonal antibodies were successfully prepared. Based on these antibodies, different CSF tau profiles in Western blots were observed between the groups of probable CJD and non-CJD. A disease-specific tau band emerged in the CSF samples from probable sporadic CJD, which may supply a new biomarker for screening sporadic CJD.

## Introduction

Creutzfeldt-Jakob disease (CJD) is a rapidly progressive and ultimately fatal disorder of the central nervous system believed to be caused by prion [1]. The clinical syndrome of CJD include rapidly progressive dementia, myoclonus, visual or cerebellar symptoms, pyramidal or extrapyramidal signs and akinetic mutism [2]. To date, a definite diagnosis can only be made by neuropathological examination and demonstration of the pathological isoform of the prion protein (PrP^Sc^) in central nervous tissues, either at biopsy or autopsy [3]. In the patients with new-variant CJD (vCJD), a pathologic isoform was also detected in tonsils [4]. Probable or possible diagnosis of CJD, especially sporadic CJD (sCJD), can be intravitally achieved according to clinical manifestations, typical changes in EEG and the appearance or alternation of some neuronal proteins in cerebrospinal fluid (CSF) [5]. However, only immunoblot for CSF 14-3-3 is included in the diagnostic criteria [6], [7], despite the fact that the other surrogate markers also may have a high differential diagnostic potential [8].

Tau is a microtubule-associated protein capable of promoting microtubule assembly and stability in the nervous system. In brains from normal human adults, six isoforms of tau have been reported, which are encoded from a single gene with 16 exons by alternative mRNA splicing [9]. They differ from one another by the presence or absence of a 29-amino acid insertion (encoded by exon-2) or 58-amino acid insertion (encoded by exon-2 and -3) in the amino-terminal half of the protein, as well as by presence or absence of a 31-amino acid repeat (encoded by exon-10) in its carboxyl-terminal half. Based on the constitution of exon-2, -3 or -10 in tau, the mature tau proteins have various lengths, including Tau352, Tau381, Tau383, Tau410, Tau412 and Tau441. All tau isoforms contain other three common repeats in its C-terminal half, which are responsible for interacting with microtubule. Therefore, according to the repeat of exon-10 in C-terminal peptide, tau proteins can be divided into two groups, 3R-tau and 4R-tau. Based on the presence of the insertion(s) of exon-2 and/or exon-3 in N-terminus, tau proteins have three forms, namely 1N-tau, 2N-tau and 0N-tau [10], [11]. The presences of tau isoforms in human brains may vary with the age, i.e. the amounts of 3R-tau and 4R-tau are comparable in adult cerebral cortex, while 0N-tau is predominant in the developing brains [12].

Under some pathological conditions, tau protein has become the major component of the intracellular filamentous deposits, such as Alzheimer's disease (AD). Altered proportions of tau isoforms have been observed in frontotemporal dementia, Parkinsonism linked to chromosome 17 (FTDP-17) and Pick disease [11]. Promising results of the diagnostic sensitivity and specificity of tau-protein in CSF with ELISA have been reported, which has been accepted as a standard procedure in AD diagnosis and evaluation of therapy [13]. The diagnostic value of CSF tau for CJD has been widely evaluated, showing a markedly disease-relative manner [14]. However, the profiles of tau isoforms in CSF of CJD patients remain unknown, possibly due to lacking of tau isoform- or exon-specific antibodies.

In this study, human tau exon-2, -3 and -10 specific fusion proteins and various tau isoform proteins were individually expressed in *E. coli*, Using tau exon-2, -3 and -10 fusion proteins as antigens, tau exon-specific polyclonal antibodies were prepared. We confirmed that the prepared antibodies recognize and distinguish tau isoforms containing various modules of exon. Based on the prepared polyclonal antibodies, the tau profiles in CSF samples from 96 Chinese patients with probable sporadic CJD, 6 genetic CJD and 22 cases who were excluded the possibility of CJD were analyzed by Western blots. A 65 KD tau-specific band, which was recognized mostly by the antibodies against tau Exon-2 and-10, emerged specifically in a large portion of CSF samples of probable sporadic CJD patients. Our study provide the reliable human tau exon-specific polyclonal antibodies and the 65 KD tau-specific band may be used as a new biomarker for screening sporadic CJD.

## Materials and Methods

### Ethics statement

Usage of the stored human clinical samples in this study, which was one of the important clinical materials in China CJD Surveillance System, has been approved by the Ethical Committee of National Institute for Viral Disease Prevention and Control, China CDC. All signed informed consents have been collected and stored by the China CJD Surveillance Centre. Animal experiment in this study was approved by the Experimental Ethical Committee of National Institute for Viral Disease Prevention and Control, China CDC. All mice and rabbits were maintained under clean grade. Housing and experimental protocols were in accordance with the Chinese Regulations for the Administration of Affairs Concerning Experimental Animals.

### Plasmid construction

The exon-2, -3 and -10 cDNAs of human tau were obtained from human peripheral blood monocytes DNA by PCR. Primers for exon-2, -3 and -10 were synthesized based on the human tau cDNA sequences released on GenBank (NM_005910.4), with a *Bam H*I or *EcoR*I restriction enzyme site at the 5′-terminus, respectively (Table 1). To construct different human tau isoforms, overlapping PCR was performed using pET-17b-tau441 [15] or pGEX-2T-tau352 [16] as the templates. The primers used in overlapping PCR contained a *Bam H*I site in the forward primers and a *Kpn*I site in the reverse primers. PCR amplification was performed at the condition of denaturing at 94°C for 30 sec, annealing at 55°C for 30 sec, extending at 72°C for 1 min, totally 30 cycles. Briefly, to construct human tau isoforms with 381 and 410 amino acids, two PCR amplifications were conducted with the primer mixture of Tau_N_ and E2-down, or E3-down, using pET-17b-Tau441 as the template, as well as the mixture of L_E2-352_ or L_E3-352_ and Tau_C_, using pGEX-2T-Tau352 as the template. After purification, two PCR products were annealed and the full-length sequences of tau381 and tau410 were amplified with primer Tau_N_ and Tau_C_. To construct tau isoform with 383 amino acids, two PCR amplifications were conducted with the primer mixture of Tau_N_ and L_E10-352_, using pGEX-2T-Tau352 as the template, as well as the mixture of E10-up and Tau_C_, using pET-17b-Tau441 as the template. After purification, two PCR products were annealed and the full-length sequence of tau383 was amplified with primer Tau_N_ and Tau_C_. To construct tau isoform with 412 amino acids, two PCR amplifications were conducted with the primer mixture of Tau_N_ and E2-down, using pET-17b-Tau441 as the template, as well as the mixture of L_E2-352_ and Tau_C_, using pET-17b-Tau383 as the template. After purfication, two PCR products were annealed and the full-length sequence of tau412 was amplified with primer Tau_N_ and Tau_C_. The four human tau isoforms were cloned into plasmid pMD18-T. After sequence verification, various isoform sequences were released from cloning vectors pET-17b-Tau441 or pGEX-2T-Tau352 and subcloned into pQE30 vector, generating prokaryotic expression recombinant plasmids pQE30-tau352, pQE30-tau381, pQE30-tau383, pQE30-tau410, pQE30-tau412 and pQE30-tau441.

**Table 1:** The primers used for the constructions of various recombinant plasmids.

Name	Sequences (from 5′ to 3′)	Enzyme
tau2-up	*GGATCC* GCACCCATGGCAGAAGGAGGAG	BamH1
tau2-down	*GAATTC* TCACCGCCTCGGCTTGTCACAT	EcoRI
tau3-up	GGATCCGCACCCATGGCAGAAGGAGGAG	BamHI
tau3-down	GAATTCTCACCGCCTCGGCTTGTCACAT	EcoRI
tau10-up	GGATCCGCACCCATGGCAGAAGGAGGAG	BamHI
tau10-down	GAATTCTCACCGCCTCGGCTTGTCACAT	EcoRI
TauN	CGGGATCCATGGCTGAGCCCCG	BamHI
E2-down	TTCCGCTGTTGGAGTGCTCTTAG	No
E3-down	TGTGGTTCCTTCTGGGATCT	No
L1-E10	AAGATCCAGCTTCTTATTAATTATCTGCACCTT CCCGCCTCCCGGCTGGTGCTTCAGG	No
TauC	GGGGTACCTCACAAACCCTGCTTG	Kpn I
LE2-352	AGCACTCCAACAGCGGAAGCTGAAGAAGCA GGCATTGGAGACA	No
LE3-352	ATCCCAGAAGGAACCACAGCTGAAGAAGCA GGCATTG	No
E10-up	GTGCAGATAATTAATAAGAAGCTGGAT	No

### Expression and purification of recombinant protein

The recombinant prokaryotic proteins tagged with GST were bacterially expressed in *E. coli* BL21(DE3) and purified with Glutathione Sepharose 4B (GE Healthcare, USA) according to the protocol described in our previous study [17]. The recombinant prokaryotic proteins tagged with six histidines were bacterially expressed in *E. coli* M15 and purified with affinity chromatography of Ni-NTA agarose (GE Healthcare, USA) according to the protocol described elsewhere [18]. Briefly, the bacteria transformed with individual expressing plasmids were grown to an OD_600_ of 0.5–0.6 and induced with isopropyl-D-thiogalactoside (IPTG) at a final concentration of 1 mM. Cells were harvested by centrifugation and resuspended in 0.01 M PBS, pH 7.4, with 1 mM phenylmethylsulfonyl fluoride (PMSF) as a protease inhibitor. Lysozyme was added to a final concentration of 2 mg/ml, and cells were lysed by incubation for 30 min and treated with sonication 24 times with power of 400 W at 10 s intervals. To obtain purified proteins, the soluble cell lysate was incubated with Glutathione Sepharose 4B or nickel-NTA agarose and stirred at 4°C overnight. Fusion proteins were eluted according to the manufacturer's protocols. The purities of the purified proteins were verified by SDS-PAGE. The specificity of the purified fusion proteins were evaluated by Western blotting with the anti-GST mAb (Tiangen Tech, China).

### Preparation and purification of mouse and rabbit anti-GST-E2, E3, E10 antisera

Antibodies against exon-2 and exon-3 of human tau were raised in rabbits and that against exon-10 was raised in mice using the purified GST-E2, GST-E3 and GST-E10 fusion proteins as antigens. Briefly, the rabbits and mice were subcutaneously injected respectively with 0.8 mg and 0.1 mg antigens mixed with an equal volume of Freund's complete adjuvant. Two weeks later, 0.5 mg and 0.08 mg antigen mixed with an equal volume of Freund's incomplete adjuvant were subcutaneously given, respectively, for a total of four-time immunizations. Three days after the last injection, the blood was taken from the carotid artery of rabbits and the hearts of mice. The whole IgG from rabbits and mice were purified by Protein A/G following the manufacturer's instructions (Sigma, USA/GE Healthcare, USA), respectively.

### Purification of human tau exon-specific antibodies

To prepare human tau exon-specific antibodies, CNBr-activated Sepharose 4B (GE Healthcare, USA) coupled with GST protein was prepared. 5 mg GST protein was dissolved in the coupling buffer (0.5 M NaCl, 0.1 M NaHCO_3_, pH 8.3). 0.3 mg freeze-dried powder of CNBr-activated Sepharose 4B was swelled and washed with 1 mM HCl (pH 2.5) in order to wash away the additives and preserve the activity of the reactive group, which otherwise hydrolyzed at high pH. Rotating the mixture end-over-end at room temperature for 1 h or at 4°C overnight. After washing away excess GST protein with 5 volumes of coupling buffer, the mixture was transferred to 0.1 M Tris-HCl buffer, pH 8.0 and incubated at room temperature for 2 h. The mixture was subsequently washed with at least three cycles of the buffers with alternating pH values. Each cycle consisted of a wash with 0.1 M acetate buffer, pH 4.0 containing 0.5 M NaCl followed by a wash with 0.1 M Tris-HCl, pH 8.0 containing 0.5 M NaCl. To remove the anti-GST component from the prepared antisera, the whole IgG from rabbits or mice were mixed with CNBr-GST agarose at the ratio of 1∶4 and incubated at 4°C overnight. The mixtures were centrifuged at 450 g for 3 min and the supernatant was collected as the purified GST-free antibody.

### ELISA

To test the specificity and sensitivity of purified human tau exon-specific antibodies, an indirect ELISA was established. Briefly, various GST-tau fusion proteins as well as GST protein were dilute to a final concentration of 2 µg/ml in PBS and coated onto 96-well ELISA plates. After blocking with 200 µl 1% BSA in PBST (phosphate buffered saline, pH 7.6, containing 0.05% Tween-20), 100 µl of prepared antibodies gradient diluted in blocking buffer were added and incubated at 37°C for 2 h. The plates were washed with PBST for three times and 100 µl of 1∶15000-diluted HRP-conjugated secondary antibody were subjected into each well at 37°C for 2 h. Bound antibody was detected using 3, 3′, 5, 5′-tetramethylbenzidine (TMB) (Sigma, USA). Absorbance was measured at 450 nm after terminating the reaction by addition of 2 M H_2_SO_4_. The titration of the purified antibodies was evaluated following the rule that positive limit is determined as *P/N* value≥2.1.

### Clinical samples

Totally 96 CSF samples from the patients diagnosed as “probable CJD”, 6 samples from the patients of “genetic or familial CJD” and 22 samples from the patients who were not fulfill with criteria for probable or possible CJD as the control subjects were included for this study. All patients were referred by the China CJD Surveillance Centre and the diagnoses of CJD were made according to WHO CJD diagnostic criteria. The criteria were described as following. Probable CJD: Patients with a rapidly progressive dementia of less than 2 years' duration, periodic sharp-wave complexes in the EEG or a positive test for 14-3-3 protein, and two of the following: myoclonus, visual or cerebellar symptoms, pyramidal or extrapyramidal signs and akinetic mutism. Possible CJD: Patients fulfilling the preceding criteria but without typical EEG abnormalities or a positive test for 14-3-3 protein. CSF samples were obtained by standard clinical procedures and were free of blood contamination.

The median ages at onset of the probable CJD and control patients were 60- (range: 33–76) and 58-year-old (range: 43–88), respectively. No age and gender differences were observed between probable CJD and control patients. Typical periodic sharp-wave complexes in EEG were observed in 57 out of 59 (94.9%) probable CJD patients, and in 2 out of 10 (20%) control patients, having significantly higher EEG positive rate than control (*P*<0.01). The positive rates of CSF 14-3-3 in probable CJD cases and control were 80.2% and 45.5%, with significant difference (*P*<0.01). Most patients were Met/Met in *PRNP* codon 129, except one Met/Val in one probable CJD and one in control. The main features of the probable CJD and control cases were summarized in Table 2.

**Table 2:** The main demological and clinical characteristics of the patients in the groups of probable CJD and control.

	Probable CJD	Control
Number (n)Male (n)	9658	2213
Female (n)	38	9
Median age at onset (y) (range)	60 (33–76)	58 (43–88)
Age at onset <50 y (%)	13 (13.5%)	6 (27.3%)
Age at onset 50–70 y (%)	72 (75.0%)	13 (59.1%)
Age at onset >70 y (%)	11 (11.5%)	3 (13.6%)
Codon 129 genotype		
Met-Met/total (%)	98.96	95.45
Met-Val/total (%)	1.04	4.54
Val-Val/total (%)	0	0
EEGPositive/total (%)	56/59 (94.9%)	2/10 (20%)
14-3-3Positive/total (%)	77/96 (80.2%)	10/22 (45.5%)

Additionally, CSF samples from six gCJD patients were enrolled in this study. They were one G200K (age: 63 y), one T188K (age: 58 y), two G114V (age: 45 and 26 y) familial CJDs (fCJD), one P102L Gestermann-Strauss-Shenken Syndrome (GSS, age: 58 y) and one D178N fatal familial insomnia (FFI, age: 24 y).

### Western blots

The recombinant fusion protein GST-E2, GST-E3 and GST-E10 were separated in 15% SDS-PAGE, the recombinant tau isoforms and human CSF samples were separated in 10% SDS-PAGE, respectively. After electro-transferred onto PVDF membranes (Millipore, USA) using a semi-dry blotting system (Bio-Rad, USA), the membranes were blocked with 1% BSA in PBST at 4°C overnight. Tau specific signals were detected with 1∶5000 mAb Anti-Tau-1 (Millipore, USA), 1∶1000 rabbit-derived tau exon-specific antibody or 1∶500 mouse-derived tau exon-specific antibody, and subsequently with 1∶10000 horseradish peroxidase (HRP)-conjugated anti-mouse or anti-rabbit IgG (Thermo, USA). The reactive signals were visualized by ECL kit (PE Applied Biosystems, USA). The image of immunoblot was scanned with Typhoon (GE Healthcare, USA) and digitalized, and saved as TIF format. The protein bands were quantified by densitometry using computer-assisted software Image TotalTech (GE Healthcare, USA).

### MALDI-ToF

Sample preparation for mass spectrometry was performed automatically in Ettan Spot Handling Workstation according to the manufacturer's protocol. The purified fusion proteins were mixed with matrix solution (15 mg/ml sinapic acid) and dotted on the targeting areas. After Drying, the samples were desalted with 0.1% trifluoroacetic acid. Samples were analyzed in a time-of-flight mass spectrometer (Ettan MALDI-ToF Pro, GE Healthcare, USA) and the signals were collected with the Ettan MALDI-ToF Pro software system.

### Statistical analysis

The data were processed with SPSS16.0 statistics software, descriptive data were expressed as median (range) for continuous variables and as percent (%) for categorical variables. Characteristic bands' relative gray levels were tested for normal distribution using Shapiro-Wilk test. Analysis of continuous variables was done using the rank sum test (for two groups, Mann-Whitney U test). Spearman's Correlation test was used to test a linear correlation between positive rate of characteristic bands and that of 14-3-3 or EEG. Categorical variables were compared using the Chi-Square test.

## Results

### Preparation of human tau exon-specific antibodies

**Figure 1:**
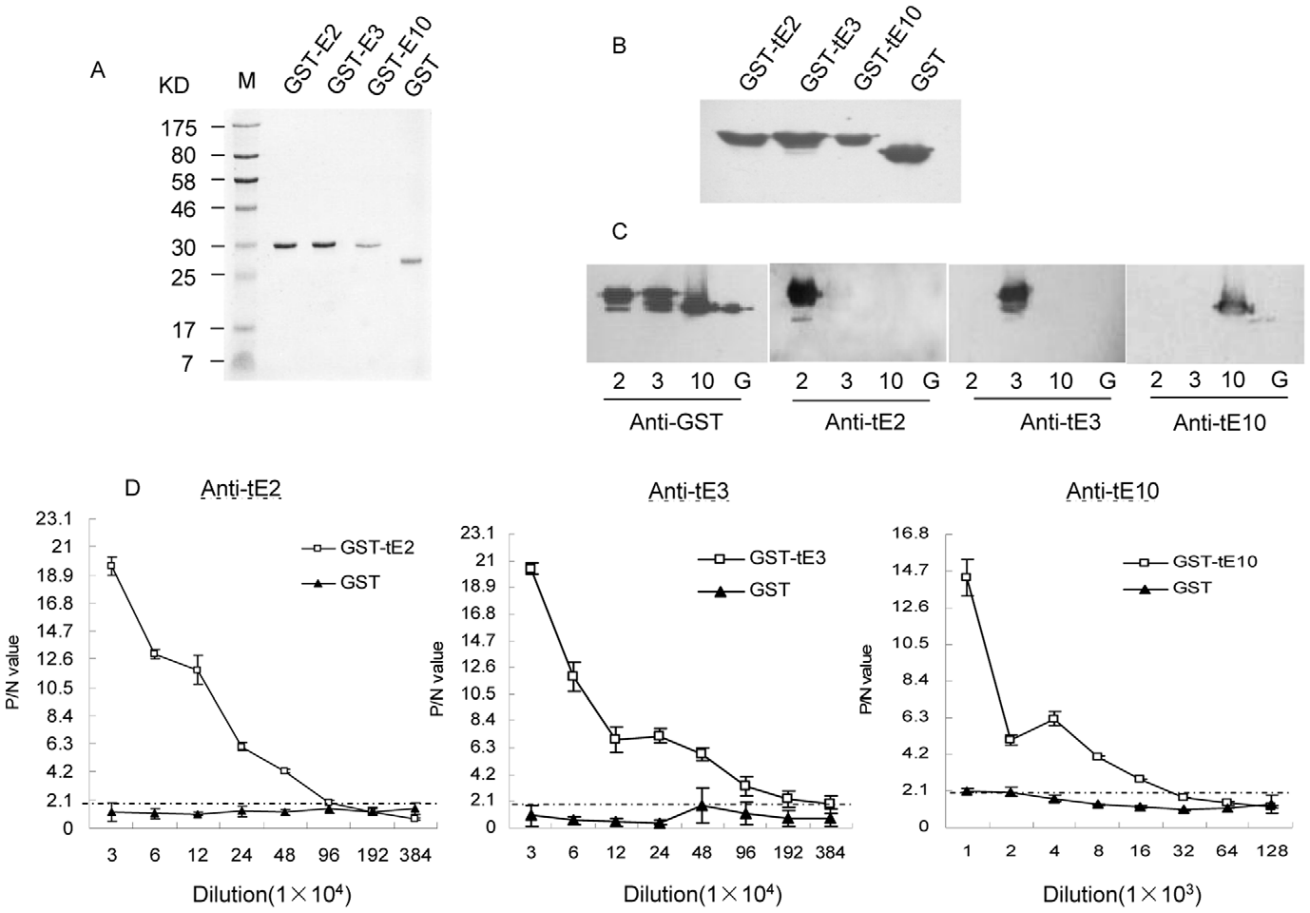
Preparations of tau exon-specific antigens and antibodies. (A) SDS-PAGE of the purified recombinant proteins GST-E2, GST-E3 and GST-E10. The proteins were separated on a 12% SDS-PAGE and stained with 0.25% Coomassie brilliant blue. Various recombinant proteins are indicated on the top. The relative molecular weights are shown on the left. (B) Identification of the recombinant proteins GST-E2, GST-E3 and GST-E10 by Western blots with GST specific antibody. Various recombinant proteins are indicated on the top. (C) Specificity assays of the purified human tau exon-specific antibodies (Anti-tE2, Anti-tE3, and Anti-tE10) by Western blots. 2: GST-E2; 3: GST-E3; 10: GST-E10; G: GST. A commercial GST antibody is used as the control. Various antibodies were indicated on the bottom. (D) Sensitivity assays of the purified human tau exon-specific antibodies (Anti-tE2, Anti-tE3, and Anti-tE10) by an ELISA coated with the purified tau exon proteins. Left panel: Anti-tE2; Middle panel: Anti-tE3; Right panel: Anti-tE10. X-axis shows the dilution of the antiserum and Y-axis shows the P/N values. The P/N value = 2.1 is illustrated with a dotted-line.

With a PCR protocol, the sequences encoding human tau exon-2, -3 and -10 were amplified, which were 87-, 87- and 93-bp long, respectively (data not shown). The PCR products were individually cloned into a prokaryotic expression vector pGEX-2T and the recombinant fusion proteins were expressed in soluble form and purified using affinity chromatography of Glutathione Sepharose 4B. SDS-PAGE gel revealed a single protein band around 30 KD in each preparation (Fig. 1A). Quantitative analyses of the gray values of all signals in each lane illustrated that the purities of GST-E2, GST-E3 and GST-E10 were approximately 88%, 84%, and 83%, respectively. Mass spectrometry assays revealed that the molecular weights of the purified GST-E2, GST-E3 and GST-E10 were 29719.319-, 29765.973- and 29780.629-Dalton (supplemental Fig. S1). Western blot analyses with GST-specific mAb identified a specific reactive band at the expected position in each preparation (Fig. 1B).

To prepare the specific antibody against different human tau exons, experimental rabbits and mice were immunized with the purified GST-E2, GST-E3 and GST-E10. The antisera were further purified with protein A/G purification and CNBr-GST agarose. To test the specificities of the prepared human tau exon-specific antibodies, the recombinant proteins GST-E2, GST-E3, GST-E10 and GST were employed into SDS-PAGE and reacted with the various prepared and commercial antibodies in Western blots. Fig. 1C confirmed that all the prepared rabbit-derived antibodies against tau exon-2 (Anti-tE2) and -3 (Anti-tE3), mouse-derived antibody against tau exon-10 (Anti-tE10) recognized only the corresponding immunogen, but not the other tau-exon fusion proteins or GST protein.

To test the sensitivity of human tau exon-specific antibodies, an indirect ELISA was carried out and equal amounts of the purified antigen or GST protein were coated on the plate. The results illustrated that the purified tau exon-specific antibodies induced very low cross-reaction with GST protein, even in the preparations with highly concentrated antiserum (Fig. 1D). With the rule of *P/N* value≥2.1, the titers of antibodies Anti-tE2, Anti-tE3 and Anti-tE10 reached to 1∶480000, 1∶1920000 and 1∶480000, respectively (Fig. 1D). It suggests that the prepared human exon-specific antibodies have reliable specificity and sensitivity.

### Tau exon-specific antibodies specifically recognized the corresponding recombinant tau isoforms

**Figure 2:**
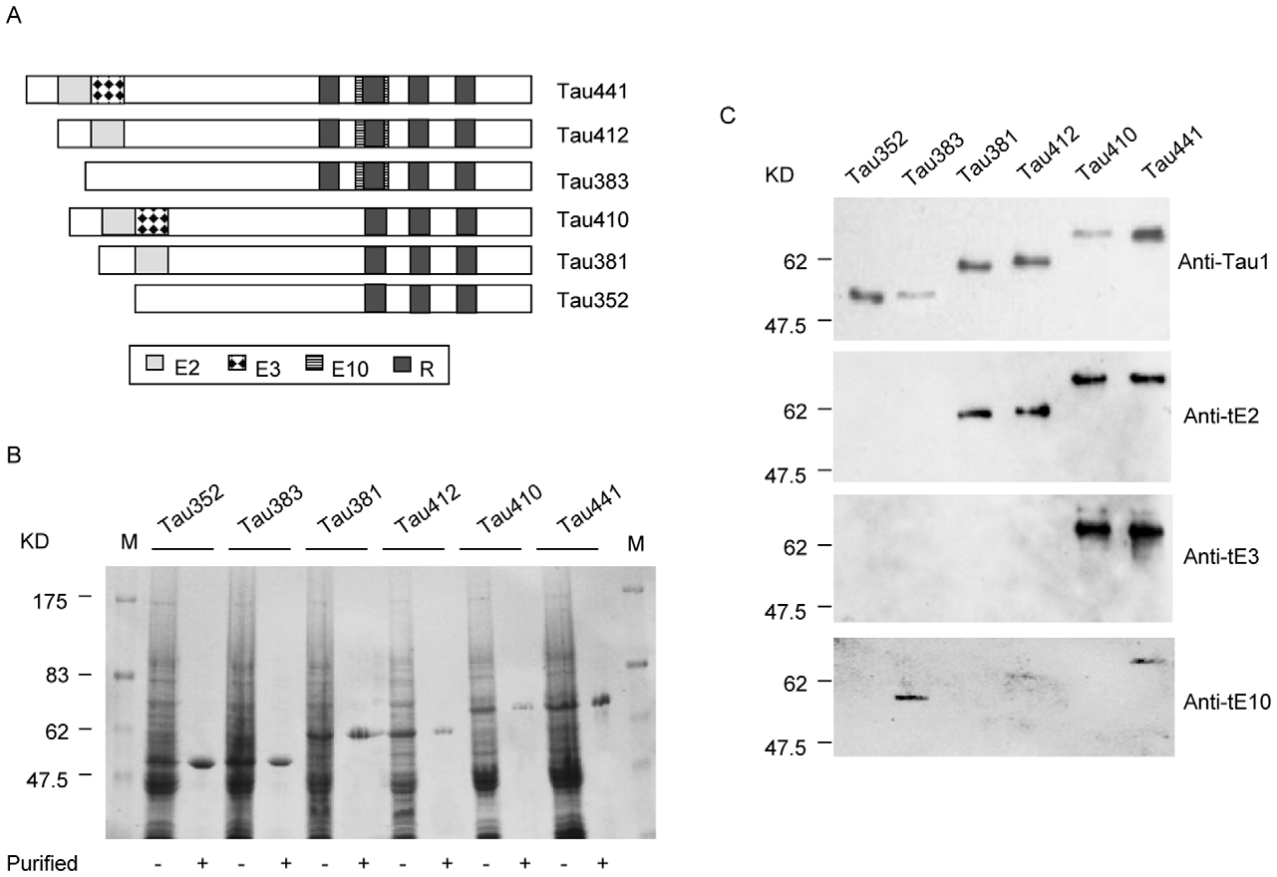
Immunoreactivities of the tau exon-specific antibodies with six recombinant human tau isoforms. (A) Schematic representation of six human tau isoforms. (B) SDS-PAGE of the purifications of six expressed tau isoforms in *E. coli*. The lysates of bacteria and the purified proteins were separated on a 12% gel and stained with 0.25% Coomassie brilliant blue. (C) Western blots of the prepared antibodies with six recombinant human tau proteins. A commercial tau mAb Anti-Tau1 is used as the control. The blots with various antibodies are indicated on the right. Different tau isoforms are shown on the top. The relative molecular weights are shown on the left.

To confirm whether prepared tau exon-specific antibodies could recognize the tau isoforms which contain relevant exons in the context of full-length protein, sequences encoding six human tau isoforms were constructed (Fig. 2A). After purified with affinity chromatography of nickel-NTA agarose, the recombinant proteins were evaluated with SDS-PAGE. Clear single band was observed in each preparation and the sizes were from 48 to 67 KD (Fig. 2B). Western blot analyses with commercially tau-specific mAb Anti-Tau1 showed specifically reactive band at the expected position in each preparation of the eluted fraction (Fig. 2C).

Six recombinant tau proteins were blotted onto PVDF membranes and incubated with three tau exon-specific antibodies, respectively. Western blots revealed that four isoforms containing exon-2 region (Tau-381, -412, -410 and -441) reacted with Anti-tE2, two containing exon-3 region (Tau-410 and -441) reacted with Anti-tE3 and three containing exon 10 region (Tau-383, -412 and -441) reacted with Anti-tE10. No cross-reaction was observed among three tested antibodies in our experimental condition, indicating that the exon-specific antibodies can recognize the relevant tau isoforms in the context of full-length protein with a reliable specificity.

### A CJD-related tau signal (Band-A) was repeatedly identified in the CSF from the patients with probable CJD

**Figure 3:**
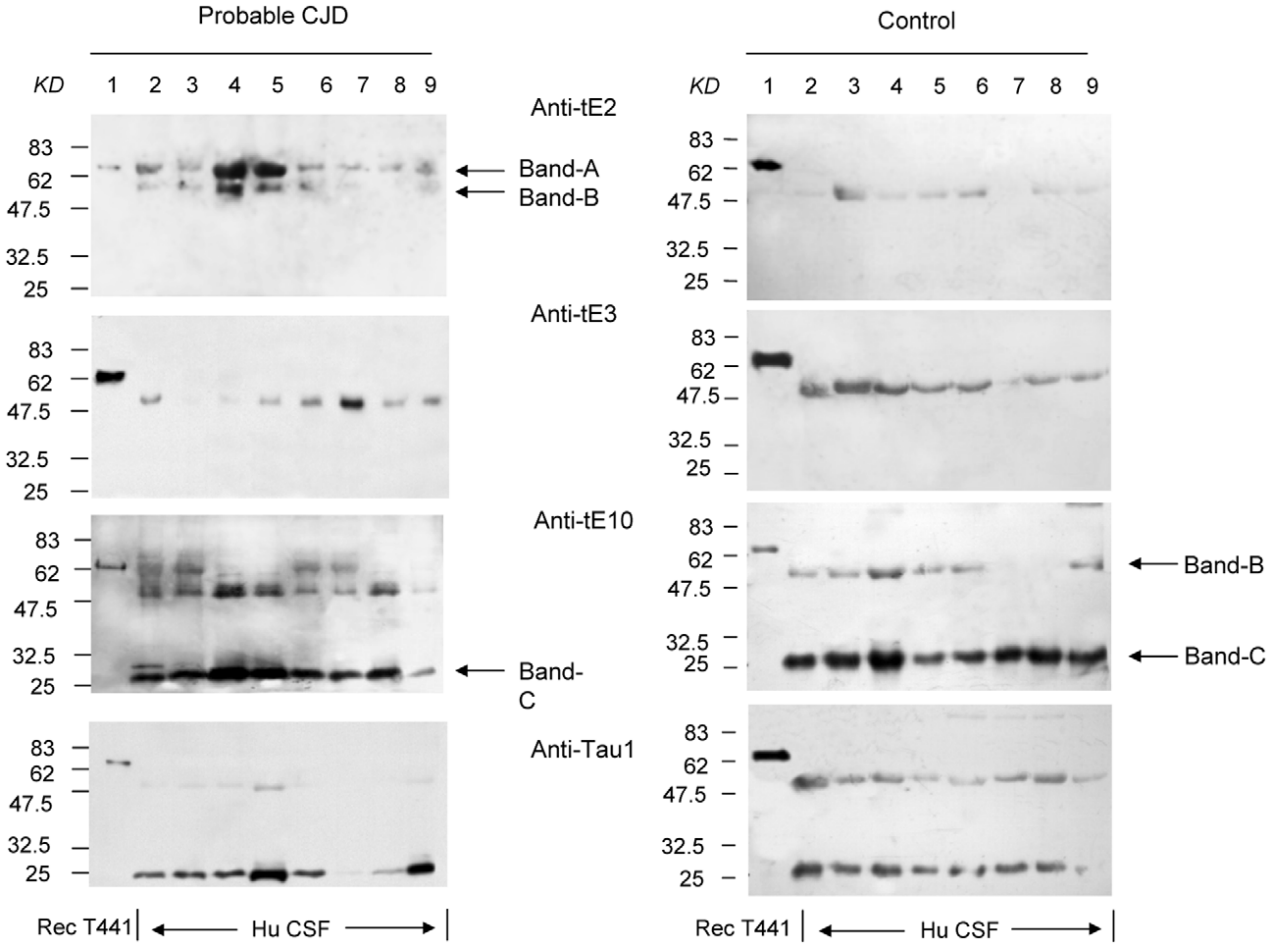
The reactive profiles of tau in CSF samples from probable CJD patient and control in Western blots. Lane 1: recombinant human tau protein Tau441 as a marker; Lane 2–9: individual CSF samples. The panels from top to bottom are the reactions with Anti-tE2, Anti-tE3, Anti-tE10 and Anti-Tau1, indicated in the middle. Left panels are CSF samples from probable and right panels are the ones from non-CJD. Tau-specific reactive bands (Band-A, -B and -C) are marked by arrows. The relative molecular weights are shown on the left.

To determine the reactive profiles of the prepared antibodies with tau in CSF, human CSF samples collected from probable CJD and non-CJD cases were employed into the Western blots reacting with the three prepared tau exon-specific antibodies and a commercial tau mAb Anti-Tau1. In general, three main reactive bands were detected in human CSF specimen, with the molecule weights of 65 KD (Band-A), 53 KD (Band-B) and 25 KD (Band-C), approximately (Fig. 3). Interestingly, the profiles of tau in CSF differed among the reactions of various tau antibodies, as well as between probable CJD and control (Table 3). Band-A was observed only in the CSF samples of probable CJD patients, but not in control samples (P<0.01). Calculations of the positive numbers of Band-A in each reactive group revealed that 38.5% samples (37/96) with Anti-tE2, 24% samples (23/96) with Anti-tE10 and 7.3% samples (7/96) with Anti-tE3 were positive, while none of the tested CSF samples showed the positive Band-A when reacted with mAb Anti-Tau1. Band-B was the most recognizable signal in each reactive group, that more than 80% tested samples in the reactions with Anti-tE2, Anti-tE3 and more than 70% in Anti-tE10, and roughly 20% samples with Anti-Tau1 showed positive Band-B. There were no statistic differences in the positive rates between the groups of probable CJD and control in the preparations of the three tau exon-specific antibodies, despite that the control group showed slight higher positive rates than the probable CJD. Contrarily, the reactions with mAb Anti-Tau1 identified significantly higher positive rate of Band-B in the control group (P<0.05). Band-C was mostly detected in the reactions with Anti-tE10 and Anti-Tau1, but infrequently in that with Anti-tE2 and not with Anti-tE3. Only the reactions of Anti-tE10 revealed significantly higher positive rate of Band-C in the control (P<0.05). Those data suggest that the CSF tau profiles in Western blots are different between the groups of probable CJD and non-CJD, in which Band-A that is frequently recognized by Anti-tE2 and Anti-tE10 seems to be related to CJD.

**Table 3:** Comparison of the positive rates of the tau-specific bands with different antibodies between the groups of probable CJD and control in Western blots.

Antibody	Band	Probable CJD(n)	Control(n)	p
Anti-tE2	Band-A*	38.5% (37/96)	0% (0/22)	0.0004
	Band-B	83.3% (80/96)	95.5% (21/22)	0.19
	Band-C	4.2% (4/96)	13.6% (3/22)	0.119
Anti-tE3	Band-A	7.3% (7/96)	0% (0/22)	0.345
	Band-B	85.4% (82/96)	100% (22/22)	0.069
	Band-C	0% (0/96)	0% (0/22)	-
Anti-tE10	Band-A*	24.0% (23/96)	0% (0/22)	0.007
	Band-B	70.8% (68/96)	90.9% (20/22)	0.051
	Band-C§	70.8% (68/96)	100% (22/22)	0.004
Anti-Tau1	Band-A	0% (0/96)	0% (0/22)	-
	Band-B§	20.8% (20/96)	77.3% (17/22)	0.0000003
	Band-C	80.2% (77/96)	81.8% (18/22)	1

*significantly higher than control group (P<0.01).

§ *significantly lower than control group (P<0.01).*

To determine the signal intensities of the tau-specific bands in Western blots in distinguishing probable CJD and non-CJD, the relative gray values of Band-B and -C in every blot of each CSF sample were counted after equilibrated with the gray value of the recombinant protein tau441 that was recognizable by all used tau antibodies. The median relative gray values of each tau-specific band reacted with each antibodies in the groups of probable CJD and control were calculated (supplemental Table S1). No statistical difference in the median relative gray values of Band-B or -C was observed between the groups of probable CJD and control, regardless of the presence or absence of positive Band-A.

### Presence of Band-A in CSF was relative to the positive 14-3-3 in CSF and typical change in EEG

To determine the possible relationship between the appearances of CSF tau-specific bands and the main demological and clinical features, the positive rates of the tau bands were analyzed based on several essential elements. No remarkable difference was found when linked with gender and the onset ages (younger than 50 y, between 50 and 70 y, elder than 70 y) of the patients (data not shown). To address the correlation with protein 14-3-3 in CSF, all 118 patients who had 14-3-3 results were grouped into 14-3-3 positive (87 cases) and negative (31 cases). More numbers of Anti-tE2-produced Band-A were detected in 14-3-3 positive group, showing a statistic difference (P<0.05) compared with that of 14-3-3 negative one (Table 4). Others, including Anti-tE3- and Anti-tE10-produced Band-A, Band-B and Band-C, were not associated with the emergence of 14-3-3 in CSF. To determine the relation with the typical abnormality in EEG, 69 patients who received EEG examinations were grouped into EEG positive (58 cases) and negative (11 cases). More numbers of Anti-tE2-porduced Band-A were found in EEG positive group, revealing a significant difference (P<0.05) compared with that of EEG negative one (Table 4). In line with the results of 14-3-3, the appearances of the rest tau-specific bands had no relation with the typical abnormality in EEG. It may emphasize again that emergences of Band-A in CSF, especially Anti-tE2-produced Band-A, have comparable significance as the presence of 14-3-3 in CSF and the typical change in EEG for CJD.

**Table 4:** Comparison of the positive rates of the tau-specific bands in different Western blots between different groups.

Antibody	Band	14-3-3 (+)	14-3-3 (−)	p	EEG (+)	EEG (−)	P
Anti-tE2	Band-A*	36.8% (32/87)	16.1% (5/31)	0.030	36.2% (21/58)	0% (0/11)	0.0145
	Band-B	83.9% (73/87)	90.3% (28/31)	0.553	86.2% (50/58)	100% (11/11)	0.338
	Band-C	4.6% (4/87)	9.7% (3/31)	0.377	5.2% (3/58)	18.2% (2/11)	0.177
Anti-tE3	Band-A	6.9% (6/87)	3.2% (1/31)	0.674	1.7% (1/58)	0% (0/11)	1
	Band-B	87.4% (76/87)	90.3% (28/31)	1.000	86.2% (50/58)	100% (11/11)	0.338
	Band-C	0% (0/87)	0% (0/31)	-	0% (0/58)	0% (0/11)	-
Anti-tE10	Band-A	20.7% (18/87)	16.1% (5/31)	0.582	25.9% (15/58)	9.1% (1/11)	0.436
	Band-B	73.6% (64/87)	77.4% (24/31)	0.672	75.7% (44/58)	72.7% (8/11)	1
	Band-C	74.7% (65/87)	80.6% (25/31)	0.505	70.0% (40/58)	90.9% (10/11)	0.268
Anti-Tau1	Band-A	0% (0/87)	0% (0/31)	-	0% (0/58)	0% (0/11)	-
	Band-B	26.4% (23/87)	45.2% (14/31)	0.054	24.1% (14/58)	54.5% (6/11)	0.067
	Band-C	79.3% (69/87)	83.9% (26/31)	0.582	84.5% (49/58)	81.8% (9/11)	1

*statistically higher than the groups of negative 14-3-3 and negative EEG, respectively (P<0.05).

### Band-A was not detected in the CSF from six genetic CJDs

**Figure 4:**
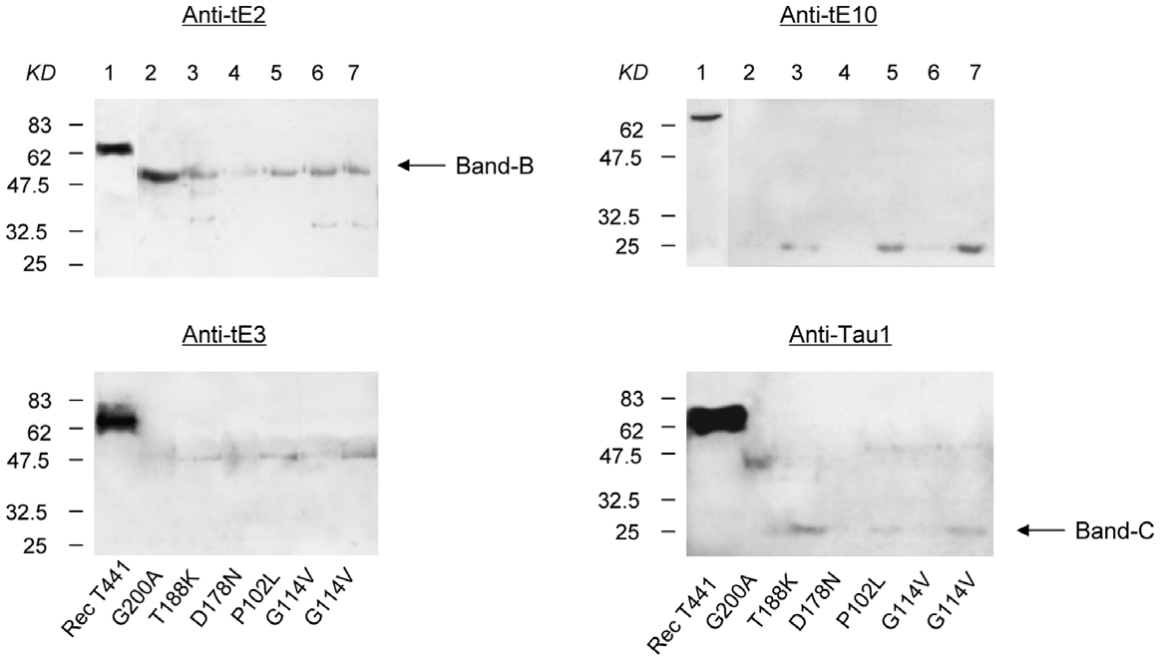
The reactive profiles of tau in CSF samples from six gCJD patients in Western blots. Various gCJD cases are indicated on the bottom. The reactions with Anti-tE2, Anti-tE3, Anti-E10 and Anti-Tau1 are shown on the top. Tau-specific reactive bands (Band-A, -B and -C) are marked by arrows. The relative molecular weights are shown on the left.

Six CSF samples from various gCJD cases were also screened for presences of tau signals by Western blots. Among them, two cases (G200A and T188K fCJDs) showed positive 14-3-3 in CSF and two others (P102L GSS and one G114V fCJD) were positive in EEG. Incubations with tau exon-specific and full-length antibodies identified predominant Band-B in all tested samples, and less predominant Band-C in some cases. However, none of them showed Band-A in their CSF (Fig. 4). It indicates that the tau profiles in CSF of the genetic CJD are different from that of sporadic CJD, possibly implying a distinct neurological pathogenesis of genetic CJD from sporadic CJD.

## Discussion

We have successfully prepared three polyclonal antibodies specifically against the peptides encoding from human tau exon-2, exon-3 and exon-10, respectively. Although the molecular weights of the peptides encoded by those three exons are similar, the amino acid sequences of each exon peptide vary tremendously, which lead us to prepare exon specific antibodies. Several reactive bands have been detected in human CSF samples by Western blots with three prepared tau polyclonal antibodies and one commercial tau mAb and three bands are predominant. The lengths of Band-A (65 KD) and -B (53 KD) are in the range of full-length tau, likely representing two forms of full-length tau. Band-C is much smaller (25 KD), possibly suggesting a form of the truncated tau. Since the maturing process of tau protein in cells undergoes post-translational modifications, i.e. phosphorylation [19], the apparent molecular weights of tau in CSF are usually different from the expected size [20]. The main phosphorylating sites within tau are locate at the tubulin binding region, including Ser-202/Thr-205, Ser-214/Ser-212, Thr-231/Ser-235 and Ser-396/Ser-404 [15]. Those sites are relatively far from the three inserted exons. Presumably, the prepared exon-specific antibodies may recognize both phosphorylated and un-phosphorylated tau proteins.

A tau band (Band-A) emerges as a specific band in CSF samples of probable CJD patients, especially when reacted with the antibodies against the tau exon-2 and -10, indicating a disease- (even CJD-) relative manner. It highlights the possibility that tau Band-A in CSF might be another useful biomarker for CJD diagnosis. Release of tau protein into CSF is a normal metabolic process [21], however, increase of tau protein in CSF is also considered as a result of neuronal damage or death. Raised CSF tau can be detected in a serial of neurological diseases, i.e. AD, frontotemporal dementia, Lewy body dementia and vascular dementia [11]. Numerous literatures have addressed the remarkably increased CSF tau in CJD patients by ELISA, proposed as one of the diagnostic criteria for CJD [22]. Whether the increased tau in CSF of CJD patients is due to the appearance of tau Band-A is unclear. Nevertheless, our data here illustrate a distinct profile of CSF tau in CJD patients, suggesting that different tau components are released from brains into CSF during CJD pathogenesis.

In line with previous tau ELISA assays [14], the appearances of Band-A of tau in CSF showed no linkage with the onset age and gender of the tested probable CJD cases. Well correlations of positive tau Band-A with positive 14-3-3 in CSF, and with typical abnormality in EEG have been observed. Presence of 14-3-3 in CSF is usually believed as a result of neuronal damage or death [23]. EEG exhibits special changes in sporadic CJD, ranging from nonspecific findings such as diffuse slowing and frontal rhythmic delta activity (FIRDA) in early stages to disease-typical periodic sharp wave complexes (PSWC) in the middle stage and a reactive coma traces or even alpha coma in late stage [24]. Both positive 14-3-3 in CSF and PSWC in EEG are included in the World Health Organization diagnostic classification criteria of CJD [6]. Significant correlations among positive tau Band-A, positive 14-3-3 in CSF and typical abnormality in EEG emphasize again that Band-A of tau in Western blots with exon-specific antibodies is a CJD-relevant event. Positive 14-3-3 in CSF and mergence of PSWC in EEG are not CJD-exclusive [24]. In contrast, appearance of the fraction of Band-A of tau seems to be more CJD-specific. More cohort studies, especially covering the CSF samples from definitely diagnosed CJD patients, will define its significance in the diagnosis for sporadic CJD.

CSF samples from six definitely diagnosed gCJD cases in this study do not reveal Band-A in Western blots. Certainly, the sample size is too small to draw any meaningful conclusion, but it raised a question of whether the detection of Band-A is suitable for gCJD. Unlike the roles in sporadic CJD, measurements of CSF 14-3-3 and EEG usually have much less significance in the diagnosis for human gCJD [22], [24]. Increased CSF tau level in gCJD is rarely observed [22]. It may reflect a complicated pathogenic mechanism of human TSE. The potential effectiveness of polymorphism at codon 129 on the appearance of tau Band-A remains unsettled, as only one case of probable CJD shows Mel/Val heterozygous, which is common feature for Han Chinese [25].

The apparent molecular weights of recombinant tau isoforms in SDS-PAGE usually differ from expected molecular weights, ranging from 48 to 67 KD. This may be related to the unusual amino acids constitutions of the inserts and the extended configuration of tau [26], [27]. The six recombinant tau isoforms expressed in this study have also revealed the difference between the apparent and expected molecular weights, e.g. recombinant Tau383 and Tau412 showing markedly small apparent molecular weights. Since more factors may affect the phenotypes of tau proteins, e.g. posttranslational modification, proteolysis and truncated expression, the tau isoforms in bio-samples are even harder to be defined in SDS-PAGE based on the apparent molecular weights. Therefore, the immunoreactivities of tau proteins with the antibodies against the different inserted exons may supply an alternative methodology to estimate the tau components in samples.

Based on the immunoreactivity, large proportion of Band-A is the tau peptides containing exon-2 and exon-10. Some of them contain only exon-2 or exon-10, and a few contain exon-2 and exon-3. This observation indicates that a single tau reactive band in Western blot may compose of several tau isoforms that migrate at the same position in SDS-PAGE. Similar phenomenon has been observed in Band-B and -C. It highlights again the difficulty in distinguishing tau isoforms with their apparent molecular weights. Under our experimental situation the commercial mAb Anti-Tau1 failed to recognize Band-A and the reason is not clear. Anti-Tau1 recognizes the internal epitopes (amino acid 189–207, according to the numbering of the longest isoform) in the intact tau protein, and our results confirmed that it can react with all recombinant tau isoforms in Western blots. Previous study has revealed that Anti-Tau1 fails to recognize the tau proteins phosphorylated at Ser-195, Ser-198, Ser-199 and/or Ser-202 [28]. Hence, we speculate that the fraction of Band-A in CSF might be a group of phosphorylated tau proteins that are wholly or partially modified at those sites.

In summary, we raised the human tau exon-specific antibodies against the peptides encoded by exon-2, -3 or -10. A CJD-related band has been detected in CSF samples of probable CJD patients. The methodology described here could be used as another tool for CJD diagnosis, using relatively easily-collected human CSF specimen, especially for CJD diagnosis in the countries like China where the brain postmortem is rarely performed because of the tradition.

## Supporting Information

Table S1 The signal intensities of various tau-specific bands in the groups of probable CJD and control in Western blots.
(0.06 MB DOC)

Figure S1 Mass spectrometry assays of the purified recombinant GST-tau exon proteins. X-axis represents the molecular weights and Y-axis represents the signal intensity. Various recombinant proteins are indicated on the top.
(0.24 MB TIF)
