# Supplementary material for: The Prepared Tau Exon-Specific Antibodies Revealed Distinct Profiles of Tau in CSF of the Patients with Creutzfeldt-Jakob Disease
Source: PLoS One. 2010 Jul 29;5(7):e11886. doi: 10.1371/journal.pone.0011886 (PMC2912377; doi:10.1371/journal.pone.0011886)
Supplement: Table S1 — The signal intensities of various tau-specific bands in the groups of probable CJD and control in Western blots. (0.06 MB DOC) [file pone.0011886.s001.doc]

Supplemental Table 1. The signal intensities of various tau-specific bands in the groups of probable CJD and control in Western blots

|  | Antibody | Band | Probable CJD  median (min max) | Control  median (min max) | *p* |
| --- | --- | --- | --- | --- | --- |
| Band-B | Anti-tE2 | A+ | 0.7604 (0.0010 8.6084) | - | - |
|  |  | A- | 0.7007 (0.0014 7.3344) | 0.5350 (0.0049 1.3473) | 0.200 |
|  |  | total | 0.7168 (0.0010 8.6084) | 0.5350 (0.0049 1.3473) | 0.124 |
|  | Anti-tE3 | A+ | 0.4601(0.1039 0.5847) | - | - |
|  |  | A- | 0.2685 (0.0028 2.2293) | 0.2118 (0.0050 0.7824) | 0.409 |
|  |  | total | 0.2751 (0.0028 2.2293) | 0.2118 (0.0050 0.7824) | 0.340 |
|  | Anti-tE10 | A+ | 0.6903 (0.2212 1.7394) | - | - |
|  |  | A- | 0.3610 (0.0017 10.2320) | 0.3773 (0.0579 1.4714) | 0.724 |
|  |  | total | 0.4448 (0.0017 10.2320) | 0.3773 (0.0579 1.4714) | 0.735 |
|  | Anti-Tau1 | A+ | - | - | - |
|  |  | A- | 0.0865 (0.0016 0.7074) | 0.1210 (0.0031 0.7073) | 0.232 |
|  |  | total | 0.0865 (0.0016 0.7074) | 0.1210 (0.0031 0.7073) | 0.232 |
| Band-C | Anti-tE2 | A+ | 0.2817 (0.2777 0.8415) | - | - |
|  |  | A- | - | 0.0501 (0.0009 0.1462) | - |
|  |  | total | 0.2797 (0.1468 0.8415) | 0.0501 (0.0009 0.1462) | 0.057 |
|  | Anti-tE10 | A+ | 1.9779 (1.1047 5.3382) | - | - |
|  |  | A- | 0.7200 (0.0144 42.4742) | 1.7180 (0.0889 33.3316) | 0.093 |
|  |  | total | 1.0935 (0.0144 42.4742) | 1.7180 (0.0889 33.3316) | 0.146 |
|  | Anti-Tau1 | A+ | - | - | - |
|  |  | A- | 0.2982(0.0028 7.9176) | 0.2720(0.1026 0.8273) | 0.825 |
|  |  | total | 0.2982(0.0028 7.9176) | 0.2720(0.1026 0.8273) | 0.825 |

*p* value for each parameter result from Mann-Whitney U test between two groups.
