# Supplementary figures and images for: The Prepared Tau Exon-Specific Antibodies Revealed Distinct Profiles of Tau in CSF of the Patients with Creutzfeldt-Jakob Disease
Source: PLoS One. 2010 Jul 29;5(7):e11886. doi: 10.1371/journal.pone.0011886 (PMC2912377; doi:10.1371/journal.pone.0011886)

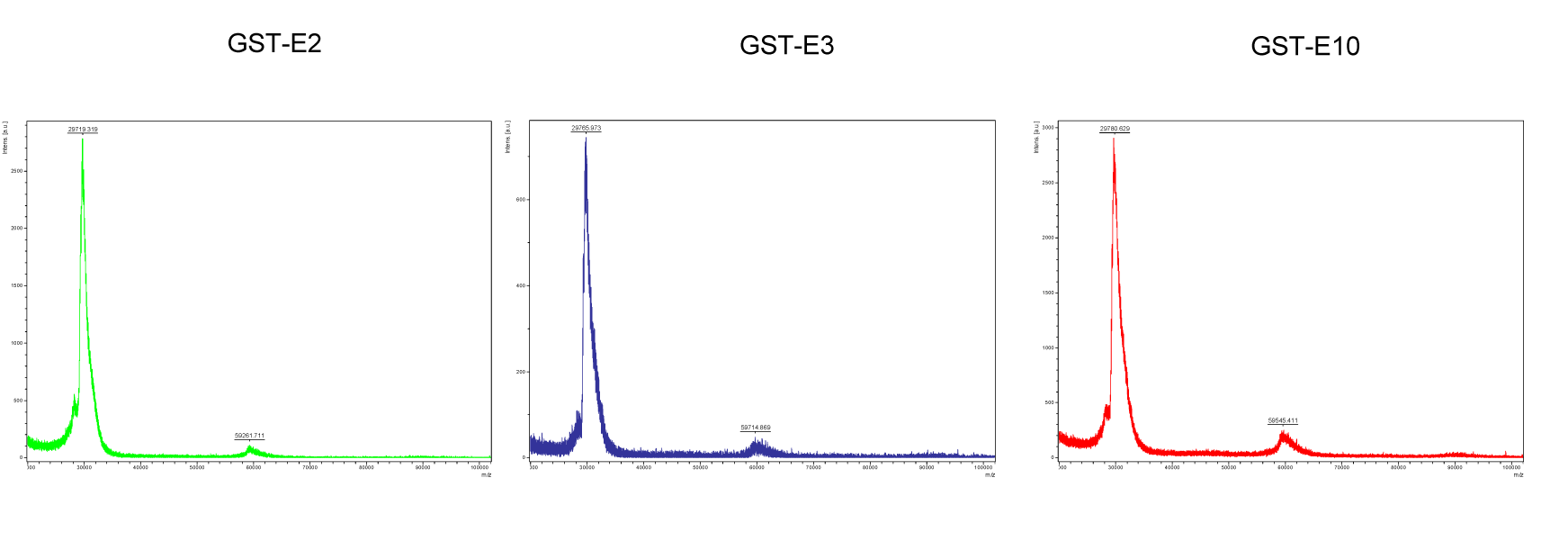

Supplement: Figure S1 — Mass spectrometry assays of the purified recombinant GST-tau exon proteins. X-axis represents the molecular weights and Y-axis represents the signal intensity. Various recombinant proteins are indicated on the top. (0.24 MB TIF) [file pone.0011886.s002.tif]
